# *Brachythecium rutabulum*, A Neglected Medicinal Moss

**DOI:** 10.1007/s10745-017-9961-y

**Published:** 2017-12-06

**Authors:** Jacek Drobnik, Adam Stebel

**Affiliations:** Department of Pharmaceutical Botany, School of Pharmacy with the Division of Laboratory Medicine, Medical University of Silesia in Katowice, Ostrogórska 30, 41-200 Sosnowiec, Poland

**Keywords:** *Brachythecium rutabulum*, *Rhytidiadelphus triquetrus*, *Homalothecium sericeum*, Materia Medica, Dressing materials, Moss, Historical botanical nomenclature

## Introduction

The search for new pharmaceuticals from naturally occurring biological materials has been guided by ethnobiological data. The investigation of folk medicine is a valuable tool in bioprospecting for pharmaceutical compounds (Costa-Neto 2002), and natural product drug development is key to the pharmaceutical industry. Over the past decade, research on medicinal plants has increasingly used historical medico-botanical texts both to study the development of pharmacopoeias as well as to identify candidate species for drug development (Staub *et al.* 2016).

The first medicinal bryophytes were noted in the first century and subsequently a relatively large number of species in the phylum Bryophyta have been recognized in medicinal usage since the sixteenth century (Drobnik and Stebel 2014, 2015). In 1600, Caspar Schwenckfeld listed six botanical names for bryophytes, which specified at least four species used as remedies in folk medicine (Drobnik and Stebel 2015). Cooper (2010) concluded that Catalogues of flora from specific European regions were published to provide local resources for the distribution and use of medicinal plants. Indigenous plants could be substituted for the exotic, often unavailable or unaffordable Materia Medica. Examples include the Harz Mountains (Thal 1588), Silesia (von Schwenckfeld 1600), Pomerania (Ölhafen 1643, 1656), and East Prussia (Loesel 1654). Since Galen’s first century works listed mostly Italian medicinal plants, these books enabled local inhabitants, including pharmacists and physicians, to harvest medicinal raw materials locally (Cooper 2010). Historical medical applications of some species bryophytes listed in these catalogues correspond with today’s pharmacological knowledge of the herb (Asakawa 2007; Asakawa *et al.* 2013; Drobnik and Stebel 2014, 2015, 2017).

Medicinal plants described in historical sources can be identified by means of a chain of synonymic botanical names (mostly pre-Linnaean), which can be cross-checked with modern knowledge of species morphology, taxonomy, phytochemistry, and ethno-pharmacology (see Drobnik and de Oliveira 2015). Information on ethno-medical and historical uses of bryophytes has been collected to target modern pharmacological research by selecting potential candidate species as medicinal plant sources (Pant 1998; Podterob and Zubets 2002; Glime 2006; Harris 2008; Bowman 2016).

Historical works have frequently provided information useful for modern medicinal therapies. For example, Adams *et al.* (2011) identified apparently lost Renaissance antimalarial remedies with proven antiplasmodial activity. The diuretic action of *Polytrichum* moss, known in seventeenth century Europe and independently used in traditional Chinese and Guatemalan medicine, was rediscovered in the nineteenth and early twentieth century (Drobnik and Stebel 2015), when *Sphagnum* moss was used for dressing wounds in 1882, and subsequently used in World War 1 simply as an absorbent. Medicinal use of *Sphagnum* peat was reported in folk medicine even earlier. Despite numerous biochemical studies of the multiple positive healing effects of *Sphagnum,* they were only finally accepted and described in the 1990s after experimental studies by the prominent British chemist, Terence J. Painter (Painter 1991, 1998, 2003; Børsheim *et al.* 2001; Stalheim *et al.* 2009; Drobnik and Stebel 2017), which facilitated the effective application of sphagnan (a *Sphagnum* herb component) for skin and wound infections.

## Study Context

While retrieving botanical data from the *Cynosura Materiae medicae* (Boecler 1731), we encountered a description of a medicinal stock named *muscus terrestris et hortensis*. Our aim was to identify one or more moss species of this stock and to compare their historical and possibly ethno-pharmacological uses with modern knowledge of the species. Because no voucher material was available in any collection, we could draw on nomenclature and ecological and pharmacological data only. First, we obtained the source texts to resolve the nomenclatures. We then addressed the historical medical applications. Since the moss(es) in question were originally mentioned as styptics, we designed an experiment to measure the absorption ratio and compare it with other known medicinal mosses in order to assess whether the historical treatments for which it was used were likely to be effective.

## Sources

We used two main sources for our research. The first, *Cynosura materiae medicae* is a multi-volume book issued, supplemented, and re-edited between 1701–1754 in Strasbourg by P. Hermann and later by J. Boecler. In the opening section, Hermann (1701) explained his choice of title, “*Cynosura* (Latin for “The Polar Star”), just like an aid to navigation, is to enable the reader to successfully complete his cruise on the ocean of life, by facilitating the choice of remedies.” The work deals with the etymology of plant names and describes their medicinal properties. *Cynosura* directs special attention to little-known plants, even those already forgotten by the eighteenth century (Drobnik 2015). A second edition of Hermann’s opus, already supplemented by Boecler, in 1731 contains a chapter entitled *Muscus terrestris et hortensis* (Boecler 1731: 444–445), entirely copied, almost word for word, from *Historia Plantarum universalis* (Bauhin 1651: 764), the primary source for the *Cynosura*.

Our second source was the *Historia Plantarum Universalis,* the major opus of Jean (Johann) Buahin (1541–1613), a Swiss botanist, was posthumously edited by J. Cherler and released in three volumes in 1650–1651. It became the most comprehensive plant encyclopaedia of the time, and was cited by botanists and pharmacists throughout the eighteenth century, including Hermann and Boecler.

## Material and methods

We found reference to each botanical polynomial mentioned in Bauhin's (1651) and Boecler's (1731) texts in the first modern taxonomical monograph on mosses (Dillenius 1741). The new names that Dillenius coined and appended to these polynomials were subsequently found in a work by Hedwig (1801). We then researched the scientific binomials Hedwig established for Dillenius' polynomials in Ochyra *et al.’s* (2003) catalogue of moss nomenclature for their currently accepted binomials. We then cross-checked the species we had identified in this manner with the description by Bauhin (1651) in terms of their compatibility with morphologies (Table 1) and assessed their ecology (habitats, distribution, and abundance) in Europe.

Bauhin's (1651) description of the plant and medicinal usages were cross-checked with modern pharmacological data in order to confirm or question appropriateness of their medicinal uses from 1651. We also consulted the *PubMed* and *ScienceDirect* databases for ethno-pharmacological data on these species.

Absorption capabilities of the moss were measured using material from the bryological herbarium at the Department of Pharmaceutical Botany of the Medical University of Silesia in Katowice (SOSN). We used specimens of *Brachythecium rutabulum* (accession number: SOSN 38635), *Rhytidiadelphus triquetrus* (SOSN 44972), and *Homalothecium sericeum* (SOSN 24285). The samples were weighed at room temperature in a relative air humidity of ~25%.

Each sample was placed in a petri dish and rinsed with water. If the excess water was not absorbed within 15 min, it was poured off and the fully moistened sample was reweighed. We thus calculated how much water accounted for 1 g of dry mass of each sample. The samples were then dried and returned to the herbarium as reference materials.

## Results

### Original Botanical Description

Original Latin text: *Muscus terrestris et hortensis*: *Omnium vulgatissimus est. hic mollicellus aspergine madentibus saxis terraeque humidiori appressus repens, mollicellus ramis longis tenuibus, foliolis acuminatis costae haerentibus, colore viridi aut ex viridi flavescentibus* (Bauhin 1651, p. 764).

Translation: Ground and garden moss. This *mollicellus* is the commonest of all on sprinkled rocks and on more humid ground, pressed, creeping, a long- and thin-twigged *mollicellus*, with acuminate leaflets, adherent with a midrib, green in color and from green to yellowish. (The Latin term *mollicellus* (from *mollis* meaning “soft”) is used for “a moss.”)

Additional ecological data are included in a description of economic values: *Muscus qui hortos et prata humecta obsidet, ita ut gramen supprimat. Martio mense cinere aboletur, sed eo quo lixivium fuerit confectum*. (That moss occupies/invades/colonizes gardens and wet meadows, (and) therefore it suppresses the grass. In March, it is exterminated with ashes, but it is similarly extinguishable with leaches.)

### Nomenclature

Boecler (1731: 444) provides four synonyms, which he copied from Bauhin (1651):

1. *Muscus querno vilissimo vilior, saxis et udis terrae glebis adnascens*. This name comes from a *locus* cited usually as *Lob. Obs. p. 643*, from *Plantarum seu Stirpium Historia* (de L'Obel 1576). The L’Obel’s name is a synonym for *Hypnum dentatum vulgatissimum, operculis obtusis* by Dillenius (1741: 295). Dillenius’ polynomial became a synonym for *Hypnum rutabulum* Hedw. (Hedwig 1801: 276), and the accepted name of this taxon is *Brachythecium rutabulum* (Hedw.) Schimp. (Ochyra *et al.* 2003).
2. *Muscus squamosus major sive vulgaris, Tourn. I. R. H. cl. 17, f. 1. g. 1.* This is correctly expressed as *Muscus squamosus major sive vulgaris* (Tournefort 1700: 553) and became a synonym for *Hypnum vulgare triangulum maximum et pallidum* (Dillenius 1741: 293). Dillenius’ name is, according to Hedwig, *Hypnum triquetrum* Hedw. (Hedwig 1801: 256), currently *Rhytidiadelphus triquetrus* (Hedw.) Warnst. (Ochyra *et al.* 2003).
3. *Muscus terrestris latioribus foliis major seu vulgaris. Raji Hist. 122.* from Ray (1686, vol. 1: 122). In Dillenius’ work, it became a synonym for his *Hypnum dentatum vulgatissimum, operculis obtusis* (1741: 295), and Hedwig established it as a binomial, *Hypnum rutabulum* Hedw. (1801: 276), today, it is *Brachythecium rutabulum* (Hedw.) Schimp. (Ochyra *et al.* 2003).
4. *Muscus terrestris et hortensis, I. B. 3. 764.* comes from a citation from Bauhin (1651, vol. 3: 764). It became a synonym for *Hypnum vulgare sericeum recurvum capsulis erectis cuspidatis* (Dillenius 1741: 323). Dillenius’ name is *Leskea sericea* Hedw., according to Hedwig (1801: 228); the name has since changed to *Homalothecium sericeum* (Hedw.) Schimp. (Ochyra *et al.* 2003).

Stokes (1812) was the only author after Boecler (1731) (and probably the only nineteenth century *Materia Medica* writer) who used some these names. He filed two species: a) *Muscus terrestris latioribus foliis major seu vulgaris. Raji Hist. 122* he considered to be *Hypnum triquetrum* (Stoke’s mistake), and b) *Hypnum vulgare sericeum recurvum capsulis erectis cuspidatis* he listed for *Leskea sericea* (correct identification).

### Original Medicinal Uses

Bauhin (1651) and Boecler (1731) reported the following usage: *Empirici hoc musco uti Joh. Bauhinus loquitur ad sistendum sanguinem utuntur, ab Ursis admoniti: hi enim quamprimum vulnerati sunt, eo sanguinem sistunt.* (Practitioners use this moss, according to Joh. Bauhin, for stemming the blood, learned from bears, which, whenever hurt, use them to stop blood.)

### Absorption Capabilities

One g of air-dried moss herb *Brachythecium rutabulum* absorbed an average of 16.1 g of water, *Rhytidiadelphus triquetrus* 10.8 g, and *Homalothecium sericeum* 11.7 g.

## Discussion

### Habitats, Ecology, and Nomenclature

To assess the accuracy of the historical descriptions, modern data are quoted (but see Table 1).

**Table 1:** Comparison of characteristics of identified moss species with the original description of *muscus terrestris et hortensis*

Characters of *muscus terrestris et hortensis* from the Bauhin's (1651) and Boecler's (1731) descriptions		*Brachythecium rutabulum*	*Rhytidiadelphus triquetrus*	*Homalothecium sericeum*
Distribution and ecology	The commonest of all […], found everywhere	true	now only regionally	now only regionally
	on sprinkled rocks	it can occur	it can grow but sporadically	frequent on rocks but on dry ones
	on more humid ground	usually so	usually so	on dry rocks and tree trunks
	it occupies/‌invades gardens	true, frequently	rather a forest species	rather not unless as an epiphyte
	[it] occupies/‌invades/‌colonizes […] wet meadows	true	rather not	false
Morphology	pressed, creeping	yes (also presenting other growth forms)	rather erect	true
	long- and thin-twigged	true	false, twigs are thick	false, twigs are thin but also short
	leaflets acuminate	true	rather not	acuminate to aristate
	[leaflets] adherent to/with a midrib	A leaf midrib (Latin: *costa*) in mosses is a microscopic structure, hardly visible without magnifying equipment. This morphological term is missing still in Dale (1737). Maybe whole arch-shaped moss stems were termed “ribs” here.		
	[leaflets] green in colour and from green to yellowish	true	rather green to whitish-green	true

**Fig. 1:**
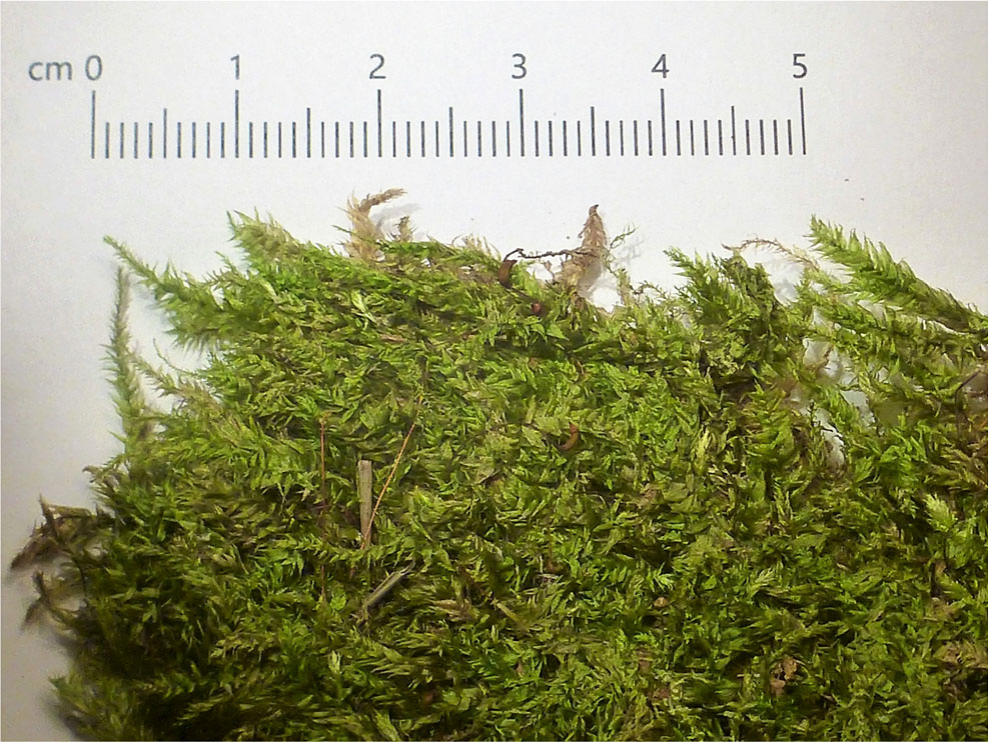
Dry *Brachythecium rutabulum* as a dressing material

*Brachythecium rutabulum* (Hedw.) Schimp. (Fig. 1) is a large moss, growing in lax, glossy, bright green or yellowish green tufts or patches. It is common in Europe and occurs in many habitats, such as soil (both in woodland and non-forest vegetation), tree boles, logs, stones, and walls (Frey *et al.* 2006). This species is frequently found in man-made habitats such as lawns in gardens, where it is regarded as an unwanted plant.

**Fig. 2:**
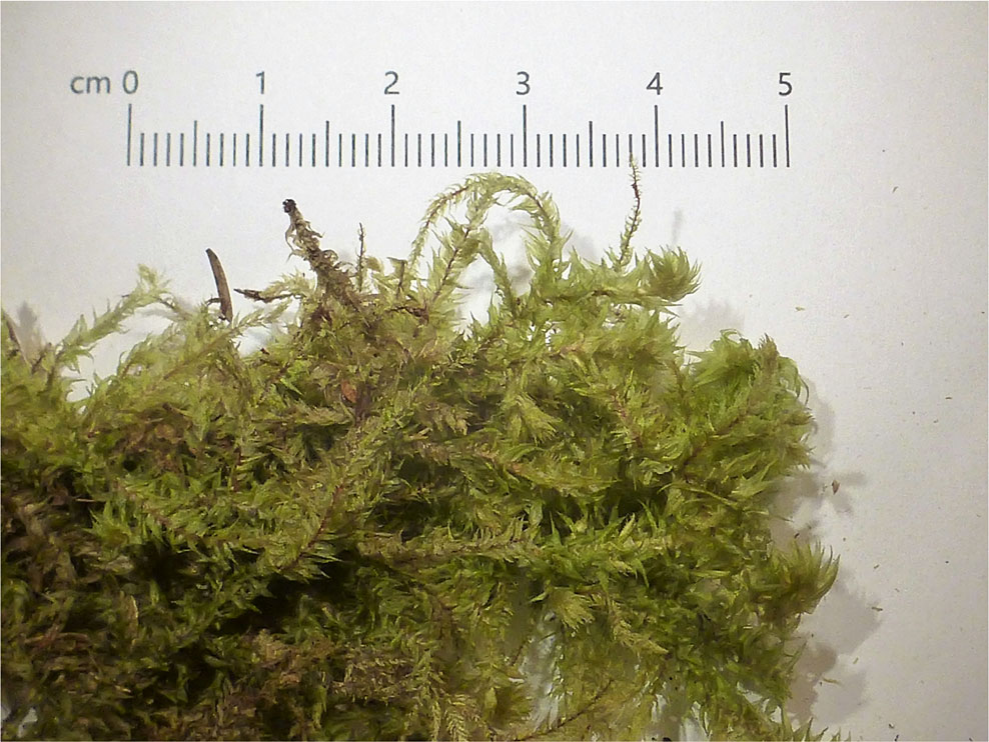
Dry *Rhytidiadelphus triquetrus* as a dressing material

*Rhytidiadelphus triquetrus* (Hedw.) Warnst. (Fig. 2) is a very robust moss, forming green, whitish green, or yellowish green tufts or patches. It grows mainly on terricolous habitats in woodlands and thickets, and is common in Europe (Frey *et al.* 2006).

**Fig. 3:**
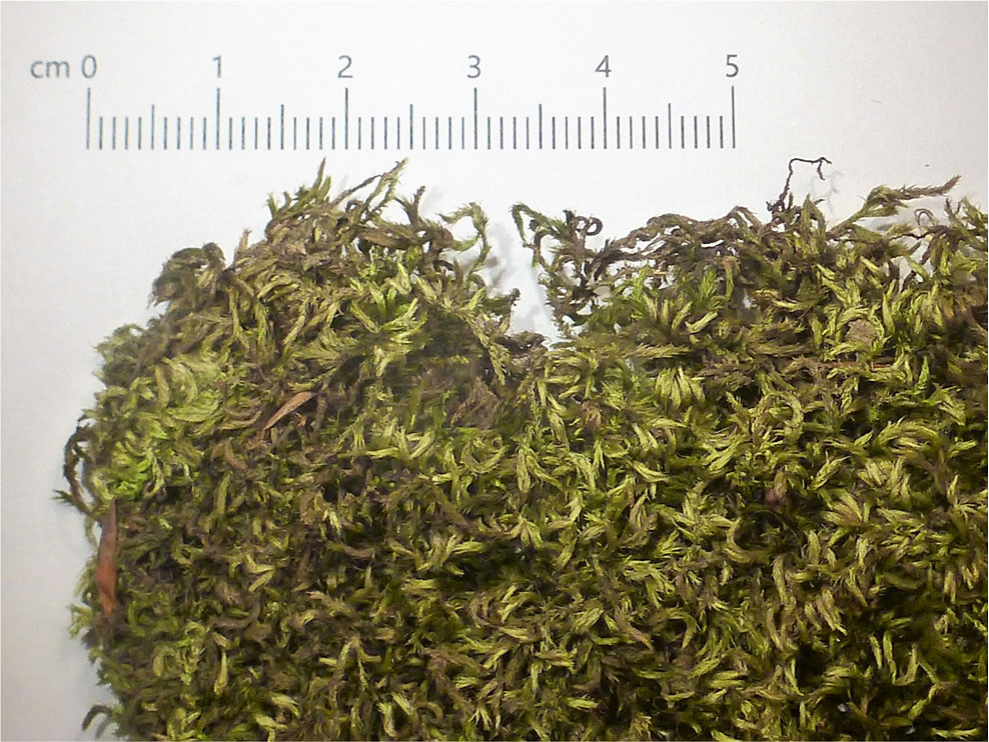
Dry *Homalothecium sericeum* as a dressing material

*Homalothecium sericeum* (Hedw.) Schimp. (Fig. 3) is moderately robust, glossy, yellowish green to golden brown, occurring in dense rough mats or patches, mainly on bark of trees and on bare rocks. Sometimes it grows on man-made habitats such as walls and roofs, and it is common in Europe (Frey *et al.* 2006).

Two pieces of information in Bauhin (1651) do not match: the statement that moss is terrestrial and that it can also occur on rocks sprinkled with water. In botany the Latin term *terrestris* traditionally refers to “growing on soil, on ground,” and in modern plant ecology this habitat is termed terricolous; a moss growing on rocks it is saxicolous (Latin: *saxatilis* or *saxicola*). Among the identified species, *Brachythecium rutabulum* and *Rhytidiadelphus triquetrus* are terricolous matching the description *muscus terrestris et hortensis* (a terrestrial and garden moss). In gardens, we should expect wet stones rather than rocky outcrops. However, compared with the other two species, *B. rutabulum* can grow on stones and it can colonize a wider range of habitats, including man-made habitats (Dierßen 2001). *Homalothecium sericeum* grows almost exclusively on tree bark or dry rock outcrops, and *Rh. triquetrus* grows mainly on the forest floor.

Regarding distribution and ecology, Boecler (1731) added the comment, *ubivis notus* (“seen everywhere”), which signifies a very common species. That this comment was added 80 years after J. Bauhin’s work leads us to assume it originated from either a herbalist’s experience (on harvesting herbal material) or perhaps only a wildlife observation. We should also note that at that time, all three mosses could be found either more or less frequently than currently.

J. Bauhin’s remark on weed control of moss and reference to its name, *muscus terrestris et hortensis,* suggests that it was a common species known by gardeners. A distinctive plant known from man-made habitats must have been named for practical purposes. Such a garden weed would most probably have been *Brachythecium rutabulum,* because this species is frequent in gardens today and is controlled by soil liming. In the past, this would have been accomplished with ashes or potash, well-known and readily available alkaline substances.

A list of polynomials (Table 2) shows nomenclatural changes between 1576 and 1801 for the species under discussion. Botanical names used by different botanists were frequently given synonyms (as depicting the same species) much later by younger botanists. In our case, J. Bauhin cited four botanical names, and thus we resolve *his* nomenclature in our text above. Any botanist could also reject some of the known names as uncertain. Almost 200 years after J. Bauhin, the prominent bryologist Dillenius collected and critically arranged *all known* bryological names (Table 2), and it is notable that some authors both before and after J. Bauhin mention certain species under even more synonyms.

**Table 2:** Polynomials treated as synonyms with their interpretation by Dillenius (1741) and his successors. Dots (●) indicate that a name is present in a given source

Name identifier in our study	Polynomial	Botanic-medical works						Dillenius’ species (polynomial) in Dillenius (1741)	Hedwig's (1801) and contemporary binomials
		(de L'Obel 1576)	(de L'Obel 1591)	(Bauhin 1651)	(Ray 1686)	(Tournefort 1700)	(Boecler 1731)		
Name 1	*Muscus querno vilissimo vilior, saxis et udis terrae glebis adnascens Lob. Obs.*	●		●			●	*Hypnum dentatum vulgatissimum, operculis obtusis*	*Brachythecium rutabulum* (Hedw.) Schimp (= *Hypnum rutabulum* Hedw.)
Name 3	*Muscus terrestris latioribus foliis major seu vulgaris. Raji Hist.*				●	●	●		
Name 2	*Muscus squamosus major sive vulgaris, Tourn. I. R. H.*					●	●	*Hypnum vulgare triangulum maximum et pallidum*	*Rhytidiadelphus triquetrus* (Hedw.) Warnst. (= *Hypnum triquetrum* Hedw.)
Name 4	*Muscus terrestris seu hortensis I. B.*			●	●		●	*Hypnum vulgare sericeum recurvum capsulis erectis cuspidatis*	*Homalothecium sericeum* (Hedw.) Schimp. (= *Leskea sericea* Hedw.)
Name 5	*Muscus 3. sive hortensis Trag.*			●				not included	
Name 6	*Muscus terrestris repens 1. sive vulgatissimus C. B. Pin.*			●				not included	
Name 7	*Muscus terrestris vulgaris Lob. Ic. [part 2 pag. 242]*		●	●				*Sphagnum palustre molle deflexum squamis cymbiformibus*	*Sphagnum palustre* L. (Linnaeus 1753)

**Fig. 4:**
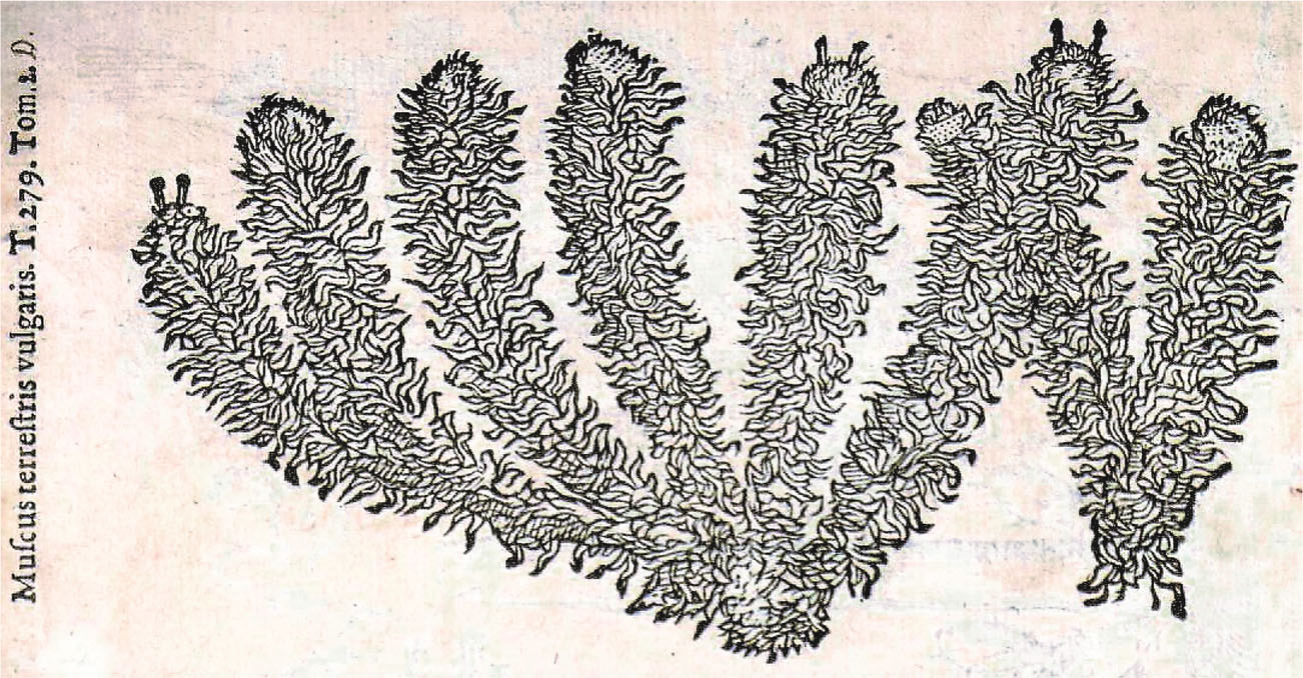
*Muscus terrestris vulgaris* (de L'Obel 1591, part 2, page 242). No description exists in the respective text-book which is de L'Obel (1576)

We can see that names no. 5–7 cited in Bauhin (1651), of which at least the seventh indicates *Sphagnum*, were excluded from synonyms by Ray (1686) and Tournefort (1700). This is illustrated from the woodcut in L’Obel (1591, vol. 2: 242), which presented a plant subtitled *muscus terrestris vulgaris* (name 7, Table 2). It had clavate (club-shaped), and densely foliate stems (Fig. 4), and highly resembles *Lycopodium clavatum* L., which in pharmacology was called *muscus terrestris,* similar to *muscus terrestris et hortensis* (Bauhin 1651), but originating from another name for *L. clavatum, muscus terrestris repens seu calvatus* (Bauhin 1623). On closer inspection, in de L'Obel (1591) woodcut, the tops of three stems reveal short pseudopodia with sporangia, typical for *Sphagnum* mosses. Ray (1686) might have noticed them and thus he no longer considered names 5, 6, and 7 synonyms for names 1, 2, 3 and 4. A herbal stock with a single known medical application (styptic) was given a scientific botanical name, *muscus terrestris et hortensis*, suggesting that this herb was named intentionally.

Based on the above discussion, we propose that the identification of *Muscus terrestris seu hortensis I. B* as *Homalothecium sericeum* as proposed by Dillenius (1741) should be considered the least probable. Moreover, the remaining synonyms (used by J. Bauhin and Ray) are identified as *B. rutabulum* or *Rh. triquetrus*. J. Bauhin wrote clearly (and Boecler repeated) that *Muscus terrestris et hortensis* is the commonest of all terricolous mosses, and Boecler added, “met everywhere.” The Latin name confirms the habitat. We should also note that in the seventeenth and eighteenth century rocky habitats were reflected in plant names by means of the adjectives *saxatilis* or *petraeus*, and stones by *lapideus*.

Another argument against *H. sericeum* is that *Brachythecium rutabulum* scored the highest absorption rate (Table 1). *Rh. triquetrus*, in second place, grows in forests but it can also be found in gardens as an ornamental. However, there are no seventeenth or eighteenth century accounts of *Rh. triquetrus* as an ornamental in Europe (Drobnik *et al.* 2016). Thus we conclude that *muscus terrestris et hortensis* is the common terrestrial moss *Brachythecium rutabulum,* although the lack of any seventeenth or eighteenth century herbarium references makes this identification a well-substantiated proposal rather than a proof.

### *Brachythecium rutabulum:* A Medicinal Moss

The differences in the water absorption ratio among the three investigated moss species result from the spatial structure of their cushions, with *B. rutabulum* being visibly the densest. Having collected data on seventeenth century uses, we assessed which of the three species is a more effective dressing for wounds, and concluded that the high absorption capacity of *B. rutabulum* make this species the most useful and easily available of all three discussed. While the Latin term, *stypticum* (blood-stemming) was understood in the eighteenth century as describing “stopping externally the blood outflow” (de Kinder and de Wint 1719: 14), in contemporary English the word *styptic* refers to the pharmacological effects of tannins; however, tannins never occur in mosses.

The absorption capacity of dry *B. rutabulum*, (16.1:1), is approximately 75% as effective as that of *Sphagnum,* which, according to Porter (1917), can absorb an average of 20 g of water per 1 g of herb. The antibacterial properties of *B. rutabulum* extracts have been recently reported by Singh *et al.* (2007), and their use has been reported in the Himalayas (Pant *et al.* 1986).

Wound dressings made of *Sphagnum* were developed in Germany in 1882 (Drobnik and Stebel 2017), but German articles “were not republished or even abstracted in English until after the (First World) war began […]” (Porter 1917). Both J. Bauhin (1651) and J. Boecler (1731) wrote about *Brachythecium rutabulum,* providing examples of the use of such moss dressings predating the use of *Sphagnum* in Germany by 231 years, and by some 263 years in England and the USA (Porter 1917).

This history would seem indicate moss dressings of the mid-seventeenth century are forgotten prototypes of *Sphagnum* dressings in World War I, and their absorptive effect on wounds must have been at least partly similar to that of *Sphagnum* (an illustration of *Sphagnum* appeared in L’Obel (1591, part 2: 242), but it was not described).

Some mosses have recently been confirmed as sources of antibacterial substances. *Brachythecium rutabulum* extract shows antibacterial activity against, for example, *Bacillus subtilis*, *Escherichia coli,* and *Staphylococcus aureus*, and antifungal activityagainst, for example, *Aspergillus flavus*, *Candida albicans,* and *Trichophyton rubrum* (Singh *et al.* 2007). The methanol extract is cytotoxic against human carcinoma cells (Ahmed *et al.* 2017) and acts as an antioxidant (Stebel *et al.* 2016). *Rhytidiadelphus triquetrus* extract shows antibacterial activity against *E. coli* (Klavina *et al.* 2015), and *Homalothecium sericeum* extract shows antibacterial activity against, for example, *Yersinia enterocolitica*, *Salmonella typhimurium*, *Bacillus subtilis,* and *Pseudomonas aeruginosa* (Çolak *et al.* 2011; Oztopcu-Vatan *et al.* 2011; Ertürk *et al.* 2015), and antifungal activity against *Aspergillus niger* and *Candida albicans* (Ertürk *et al.* 2015). Ash of burnt *Brachythecium rutabulum* and other moss species mixed with fat and honey is used as an ointment in the Himalayan region for cuts, burns, and wounds (Pant *et al.* 1986, as cited in Singh *et al.* 2007).

## Conclusions

We draw two main conclusions from this study:

1. The seventeenth century botanical name, *Muscus terrestris et hortensis,* refers to *Brachythecium rutabulum,* and thus it can be assumed that *Brachythecium rutabulum* dressings could have been used long before 1882, when *Sphagnum* was reported as used in Germany for the same purpose.
2. Based on the three species we examined in this study, the common moss *Brachythecium rutabulum* showed the highest absorption capacity for water. Descriptions from 1651 are most consistent with the ecology and morphology of this species. Based on its ecology and structure, it *was* a possible dressing material in seventeenth century Europe.

The spatial structure of *B. rutabulum* defines its physical properties and substantiates its historical use against bleeding. However, because the antimicrobial or anti-inflammatory activities have been confirmed in topical uses of both these and other mosses, the pharmacology of *B. rutabulum-*based dressings (or *B. rutabulum*-derived topical drugs) requires additional experimental studies.
